# Binding of Sperm to the Zona Pellucida Mediated by Sperm Carbohydrate-Binding Proteins is not Species-Specific *in vitro* between Pigs and Cattle

**DOI:** 10.3390/biom3010085

**Published:** 2013-01-25

**Authors:** Kazuya Takahashi, Kazuhiro Kikuchi, Yasuomi Uchida, Saeko Kanai-Kitayama, Reiichiro Suzuki, Reiko Sato, Kazunori Toma, Masaya Geshi, Satoshi Akagi, Minoru Nakano, Naoto Yonezawa

**Affiliations:** 1 Graduate School of Science, Chiba University, Chiba 263-8522, Japan; E-Mails: zag21019@gmail.com (K.T.); yasu-omi.dear@ezweb.ne.jp (Y.U.); s_kitayama@kyoritsuseiyaku.com (S.K.-K.); suzuki_016@hotmail.com (R.S.); mnakano-1359@dgs.nir.jp (M.N.); 2 National Institute of Agrobiological Sciences, Ibaraki 305-8602, Japan; E-Mail: kiku@affrc.go.jp (K.K.); 3 The Noguchi Institute, Tokyo 173-0003, Japan; E-Mails: resato@noguchi.or.jp (R.S.); toma.kb@om.asahi-kasei.co.jp (K.T.); 4 National Institute of Livestock and Grassland Science, Ibaraki 305-0901, Japan; E-Mails: geshi@affrc.go.jp (M.G.); akagi@affrc.go.jp (S.A.)

**Keywords:** glycolipid analog, glycoprotein, sperm-ligand, zona pellucida

## Abstract

Carbohydrates are candidates for the basis of species-selective interaction of gametes during mammalian fertilization. In this study, we sought to clarify the roles of sugar residues in the species-selective, sperm–oocyte interaction in pigs and cattle. Acrosome-intact porcine and bovine sperm exhibited their strongest binding affinities for β-Gal and α-Man residues, respectively. Porcine-sperm specificity changed from β-Gal to α-Man after the acrosome reaction, while bovine-sperm specificity did not. Binding of acrosome-intact and acrosome-reacted sperm decreased after trypsinization, indicating that the carbohydrate-binding components are proteins. While immature oocytes bound homologous sperm preferentially to heterologous sperm, oocytes matured *in vitro* bound similar numbers of homologous and heterologous sperm. Lectin staining revealed the aggregation of α-Man residues on the outer surface of the porcine zona during maturation. In both species, zona-free, mature oocytes bound homologous sperm preferentially to heterologous sperm. The lectin-staining patterns of the zona pellucida and zona-free oocytes coincided with the carbohydrate-binding specificities of acrosome-intact and acrosome-reacted sperm, respectively, supporting the involvement of carbohydrates in gamete recognition in pigs and cattle. These results also indicate that sperm-zona pellucida and sperm–oolemma bindings are not strictly species-specific in pigs and cattle, and further suggest that sperm penetration into the zona and/or fusion with oolemma may be species-specific between pigs and cattle.

## 1. Introduction

Mammalian fertilization requires several steps. In most species, capacitated sperm bearing an intact acrosome bind to the zona pellucida (ZP), a glycoprotein-rich layer surrounding the oocyte plasma membrane (oolemma). Once bound, these sperm undergo the acrosome reaction, which facilitates penetration. Once the acrosome-reacted sperm cell reaches the perivitelline space, it binds to the oolemma and gamete membrane fusion occurs [1,2]. Thus, two stages of binding between gametes, acrosome-intact sperm–ZP and acrosome-reacted sperm–oolemma, must occur before membrane fusion.

The ZP comprises three or four ZP glycoproteins (ZPGs). In many mammalian species, there are four ZPGs (ZP1, ZP2, ZP3, and ZP4) [3], whereas the mouse ZP comprises three ZPGs (ZP1, ZP2, and ZP3). The porcine and bovine ZPs comprise three ZPGs (ZP2, ZP3, and ZP4). In mice, ZP3 alone binds acrosome-intact sperm [4], while in pigs and cattle, ZP3 alone does not bind sperm, but instead a heterocomplex of ZP3 and ZP4 binds acrosome-intact sperm [5,6,7]. ZP4 is responsible for the porcine sperm-binding activity of the ZP3/ZP4 complex [8]. A recent study showed that ZP2 alone can bind to acrosome-intact sperm in humans [9]. Thus, sperm–ZP binding mechanisms may not be conserved in mammals.

**Figure 1:**
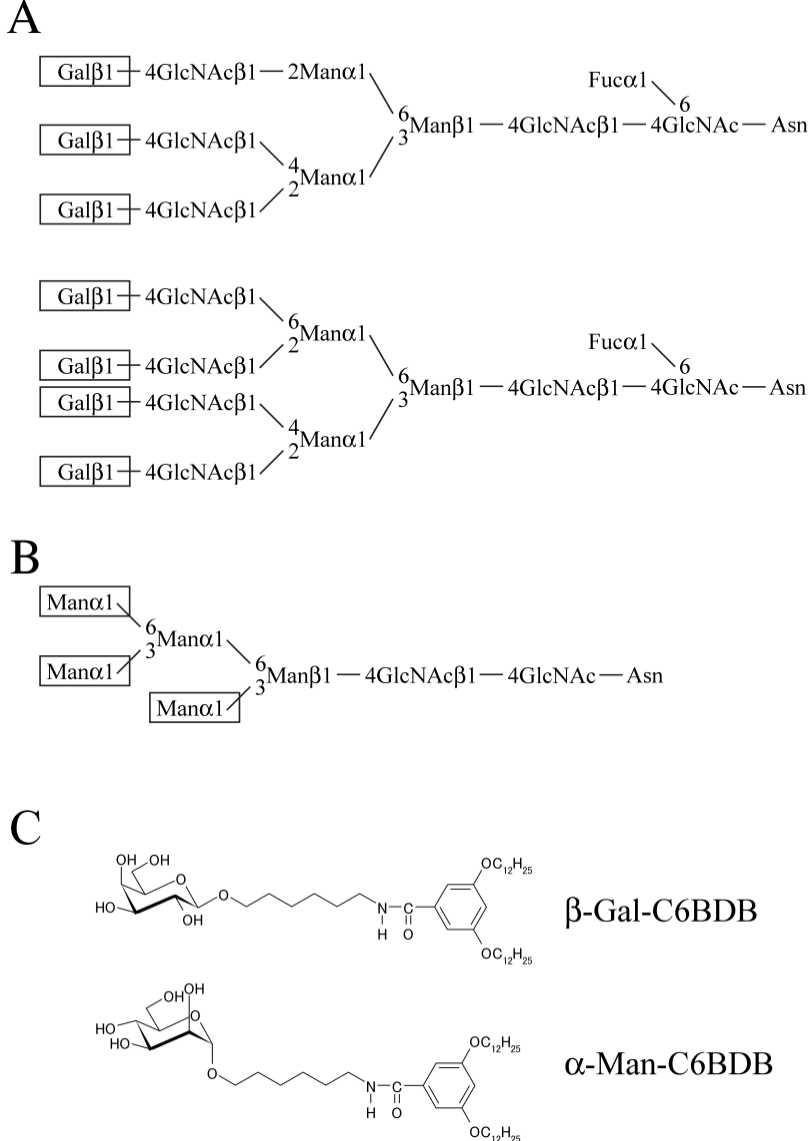
Sperm ligand carbohydrate chains of porcine and bovine zonae pellucidae and chemical structures of glycolipid analogs. The nonreducing terminal β-Gal residues (boxed) of the complex-type chains shown in **(A)** are sperm-binding sites in the porcine-zona pellucida [18].The nonreducing terminal α-Man residues (boxed) of the high-mannose-type chain shown in **(B)** are sperm-binding sites in the bovine-zona pellucida [17]. **(C)** Structures of glycolipid analogs. In addition to these two analogs, β-GalNAc-C6BDB, β-Glc-C6BDB, β-GlcNAc-C6BDB, and α-NeuAc-C6BDB were used in the present study. Nonreducing terminal β-Gal and β-GalNAc have C-3 equatorial and C-4 axial OH groups, while nonreducing terminal α-Man, β-Glc, and β-GlcNAc have C-3 equatorial and C-4 equatorial OH groups.

Studies of murine sperm–ZP binding are the most advanced among mammals. In mice, an essential role of the carbohydrate moiety of ZP3 in sperm recognition has been proposed in many studies since it was reported that *O*-linked chains of ZP3 are essential for sperm binding to ZP3 [2,4,10]. The proposed role of carbohydrate chains in sperm recognition has been rarely supported by a series of studies using transgenic mice. Furthermore, the supramolecular structure of the ZP may be necessary for sperm binding in mice [11]. As pointed out in recent reviews [12,13,14], however, conditional knockout of the *N*-acetylglucosaminyltransferase I gene in mouse oocytes reduces the sperm binding ability compared with wild-type oocytes and reduces fertility, suggesting the involvement of complex and/or hybrid type *N*-glycans in sperm recognition. The current model of sperm recognition by ZP is that both protein and carbohydrate moieties in the domain(s) of ZPGs are involved in sperm recognition [12,13,14]. More recently, it was shown that sperm–ZP binding is not necessary for fertilization of oocytes surrounded by cumulus oophorus using transgenic mice [15]. Whether this finding is applicable to mammals other than mice remains to be elucidated. In large livestock, we have shown that nonreducing terminal β-Gal residues in the tri-antennary and tetra-antennary complex-type chains of ZPGs are porcine sperm ligands, whereas nonreducing terminal α-Man residues in the high-mannose chains of ZPGs are bovine sperm ligands (Figure 1A,B) [16,17,18]. On the other hand, *O*-linked chains of porcine ZPGs also reportedly have sperm binding activity [19]. Sialic acids at the nonreducing ends of acidic *N*-linked and/or *O*-linked chains of bovine ZPGs are also involved in sperm binding [20].

The gamete molecules that participate in the sperm–oolemma interaction have been identified, including Izumo1 and CD9 [21,22]. Involvement of carbohydrates in the sperm–oolemma interaction in mice, hamsters, and humans has been reported [23,24,25]. A human sperm 65-kDa protein binds α-Man and is a candidate for an oolemma binding protein [25]. In cattle, α-Man and GalNAc inhibit sperm–oolemma fusion but do not inhibit binding [26,27]. In pigs, the involvement of carbohydrates in sperm–oolemma interaction has not yet been reported.

Several groups have investigated interspecific gamete interaction using zona-intact and zona-free oocytes isolated from rodents. These studies revealed that the ZP represents a species-specific barrier to fertilization [28,29]. However, sperm–ZP binding is not as species-specific as thought previously. Human sperm binds to the ZP of an ape (gibbon), but not to those of sub-hominoid primates (baboon, rhesus monkey, squirrel monkey) or non-primate eutherian oocytes (rabbit, mouse, hamster, guinea pig) [30,31,32]. Human sperm binds to the ZP of pigs [33]. In large domestic animals, heterologous gamete interactions between equine sperm and bovine oocytes [34] and porcine sperm and bovine oocytes [34] and an interspecific interaction between equine and porcine gametes have been reported [35]. However, these studies were not focused on the carbohydrate-binding specificity of sperm. During *in vitro * fertilization (IVF), complete species specificity is observed between heterologous gametes in pigs and cattle (Naoto Yonezawa and Minoru Nakano, unpublished data). As a first step in clarification of the mechanism of this species specificity, in this study we investigated whether structural differences in sperm ligand carbohydrates are related to sperm–ZP and sperm–oolemma interactions in pigs and cattle.

## 2. Experimental Section

### 2.1. Materials

The glycolipid analogs used for sperm binding assay are as follows: *N*-[*O*-(β-D-galactopyranosyl)-6-oxyhexyl]-3,5-bis(dodecyloxy)benzamide termed β-Gal-C6BDB, *N*-[*O*-(α-D-mannopyranosyl)-6-oxyhexyl]-3,5-bis(dodecyloxy)benzamide termed α-Man-C6BDB, *N*-[*O*-(β-D-glucopyranosyl)-6-oxyhexyl]-3,5-bis(dodecyloxy)benzamide termed β-Glc-C6BDB, *N*-[*O*-(2-acetoamide-2-deoxy-β-D-galactopyranosyl)-6-oxyhexyl]-3,5-bis(dodecyloxy) benzamide termed β-GalNAc-C6BDB, *N*-[*O*-(2-acetoamide-2-deoxy-β-D-glucopyranosyl)-6-oxyhexyl]-3,5-bis(dodecyloxy)benzamide termed β-GlcNAc-C6BDB, and *N*-[*O*-(α-*N*-acetylneuraminosyl)-6-oxyhexyl]-3,5-bis(dodecyloxy)benzamide termed α-Neu5Ac-C6BDB. The details of the synthesis of these glycolipid analogs will be reported elsewhere. Porcine and bovine ZPG mixtures were prepared from cryopreserved ovaries as described previously [16,36]. eCG (PMS 1,000 U) and hCG (Puberogen 1,500 U) were purchased from Nihon Zenyaku Kogyo (Koriyama, Japan) and Sankyo (Tokyo, Japan), respectively. Fetal bovine serum (FBS) and FSH were from Cansera International Inc. (Ontario, Canada) and Denka Pharmaceutical Co. Ltd. (Kanagawa, Japan), respectively. Medium-199 with Earle's salts, tissue culture medium (TCM)-199 with Hanks salts, Hepes and Hoechst 33342 were from Gibco Invitrogen Corp. (Carlsbad, CA), Gibco Life Technologies (Grand Island, NY), Dojindo Laboratories (Kumamoto, Japan) and Merck (Tokyo, Japan), respectively. Paraffin oil from Nacalai Tesque (Kyoto, Japan). Of seven biotin-conjugated lectins used, *Ricinus communis* agglutinin (RCA), *Triticum vulgaris* agglutinin (WGA), *Sambucus sieboldiana* agglutinin (SSA), *Maackia amurensis* agglutinin (MAM), and *Aleuria aurantia agglutinin* (AAL) were from Seikagaku Co. (Tokyo, Japan) and *Galanthus nivalis* agglutinin (GNA) and *Wistaria floribunda* agglutinin (WFA) were from EY Laboratories (San Mateo, CA). UltraAvidin-Fluorescein was from Leinco Technologies Inc. (St. Louis, MO). Penicillin G potassium, streptomycin sulfate, hyaluronidase, polyvinylpyrrolidone (PVP-40T) and pronase were from Sigma (St. Louis, MO). Pig trypsin was from Promega (Madison, WI). All other reagents were analytical grade.

### 2.2. Assessment of Porcine and Bovine Sperm Binding to Glycolipid Analogs

Binding of six types of glycolipid analog (see Figure 1C) to the wells of 96-well plates (Nalge Nunc International, Rochester, NY) reached saturation when 2.0 µg of each glycolipid analog was added to the wells as revealed by using lectins (data not shown). Binding of the porcine and bovine-ZPG mixtures reached saturation when 0.5 µg of ZPG mixture was added to the wells as revealed by using lectins (data not shown).

Six types of glycolipid analog (2.0 µg/20 µL ethanol), porcine or bovine ZPG mixtures (0.5 µg/50 µL) in phosphate-buffered saline (PBS; 150 mM NaCl, 20 mM potassium phosphate, pH 7.4), or 50 µL PBS were added separately to 96-well plates and then incubated overnight at room temperature. The wells were rinsed with 200 µL of PBS, blocked with 250 µL of 3% BSA in Tris-buffered saline (TBS; 20 mM Tris–HCl, 150 mM NaCl, pH 7.4) at 37 °C for 2 h, and then rinsed with 200 µL of Peterson's medium [37]. Porcine cryopreserved epididymal sperm and bovine cryopreserved ejaculated sperm were thawed, capacitated as previously described [6,16], and added (1 × 10^6^ cells/50 µL) to the wells coated with glycolipid analog or the ZPG mixture. To investigate the inhibitory activity of the ZPG mixture, 50-µL aliquots of porcine or bovine sperm were treated with the porcine-ZPG (1.0 µg) or the bovine-ZPG (0.6 µg) mixture, respectively, for 30 min and added to the appropriate wells. Because obtaining a large amount of ZPG mixture from bovine ovaries is difficult compared to porcine ovaries, we reduced the amount of bovine-ZPG mixture in this assay to 0.6 µg. After a 3-h incubation at 37 °C in 5% CO_2_ (porcine sperm), or at 38.5 °C in 2% CO_2 _(bovine sperm), the wells were washed three times with 250 µL of Peterson's medium. Next, 70% glycerol in PBS (100 µL) was added to each well, and the bound sperm were recovered by vigorous and repeated pipetting. The number of sperm in 0.1 µL of the suspension was quantified using a hemocytometer.

### 2.3. Assessment of Sperm Binding After Trypsin Treatment

**Figure 2:**
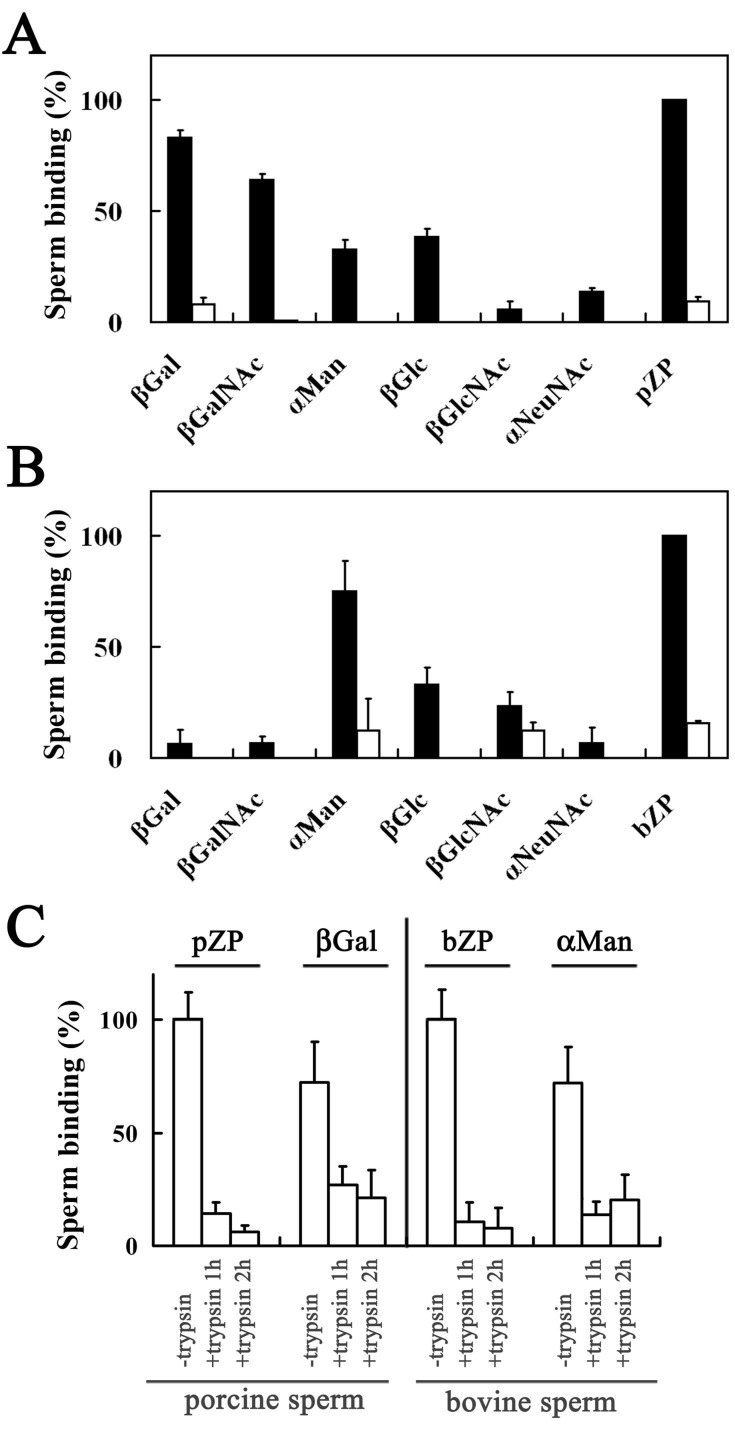
Sperm binding to plastic wells coated with glycolipid analogs and the zona protein (ZPG) mixture. The number of porcine sperm (**A**) and bovine sperm (**B**) bound to plastic wells was scored and expressed as a percentage of the number bound to the porcine ZPG mixture (pZP) in (**A**) and the bovine ZPG mixture (bZP) in (**B**), respectively. The mean number of sperm bound to the wells coated with pZP and bZP were 43 and 33, respectively, and were designated as 100%. The binding numbers of sperm not treated with the ZPG mixture were shown by solid bars. In the glycolipid analogs of βGal, βGalNAc, and pZP in (**A**) and that of αMan, βGlcNAc, and bZP in (**B**), the binding numbers of sperm treated with the respective ZPG mixture were also shown (open bars). Assays were repeated five times, and the data are shown as the mean ± SD. βGal, β-Gal-C6BDB; βGalNAc, β-GalNAc-C6BDB; αMan, α-Man-C6BDB; βGlc, β-Glc-C6BDB; βGlcNAc, β-GlcNAc-C6BDB; αNeuNAc, α-NeuNAc-C6BDB. (C) Effect of trypsinization of sperm for 1 or 2 h on the binding of porcine and bovine sperm to plastic wells. The number of trypsin-treated sperm that bound to the wells coated with pZP or β-Gal-C6BDB (βGal) and bZP or α-Man-C6BDB (αMan) were scored and expressed as a percentage of the number of sperm not treated with trypsin bound to their respective ZPG mixture.

Capacitated porcine and bovine sperm were suspended separately in BSA-free Peterson's medium (1 × 10^6^ cells/50 µL). The suspensions were incubated at 37 °C for 2 h without trypsin (–trypsin in Figure 2C). After 1 h, the suspensions were mixed with 1 µL of the same medium containing 0.5 µg of pig trypsin and incubated for an additional hour (+trypsin 1 h in Figure 2C). The suspensions were incubated for 2h with trypsin (+ trypsin 2 h in Figure 2C). After a 2-h incubation, the suspensions were centrifuged at 2000 × *g* for 1 min, and the pellets were washed once with 1 mL of medium. The porcine-sperm pellet was suspended in fresh medium at a concentration of 4 × 10^5^ cells/50 µL and added to wells coated with porcine-ZPG mixture or β-Gal-C6BDB in 96-well plates. The bovine sperm suspension (4 × 10^5^ cells/50 µL) was added to the plates coated with bovine-ZPG mixture or with α-Man-C6BDB. The incubation and quantification of bound sperm were performed as described above.

### 2.4. Collection of Porcine and Bovine Ovarian Oocytes

Porcine ovaries were obtained from prepubertal pigs at a local slaughterhouse and transported to the laboratory at 35 °C. Cumulus-oocyte complexes (COCs) were collected from follicles (3–5 mm) in TCM-199 supplemented with Hanks' salts, 10% (v/v) FBS, 20 mM Hepes, 100 IU/mL penicillin G potassium, and 0.1 mg/mL streptomycin sulfate [38].

Bovine ovaries were obtained from a local slaughterhouse, transported to the laboratory, and stored at 15 °C overnight [39,40]. After testing negative for bovine spongiform encephalopathy, the ovaries were washed in saline and processed. Follicular fluid was aspirated into syringes using an 18G-needle and transferred into plastic tubes containing TCM-199 supplemented with Hanks' salts and 3% (w/v) PVP-40T. The tubes were allowed to sit for several minutes and the supernatant was discarded. The sediment (COCs) was maintained in collection medium.

Cumulus cells were stripped from the porcine and bovine COCs by digestion with 150 IU/mL hyaluronidase for 5 min followed by gentle pipetting. These cumulus-free oocytes were used as immature oocytes. 

### 2.5. In vitro Maturation of Porcine and Bovine Ovarian Oocytes

*In vitro* maturation (IVM) was performed to prepare oocytes for fertilization according to the method described by Kikuchi *et al.* [41]. Approximately 40 porcine COCs were cultured separately in 500 μL of maturation medium, which contained modified North Carolina State University-37 solution [42], 10% (v/v) porcine follicular fluid, 0.6 mM L-cysteine, 50 mM β-mercaptoethanol, 1 mM dibutyl cAMP (dbcAMP; Sigma), 10 IU/mL eCG, and 10 IU/mL hCG, in 4-well plates (Nunclon Multidishes; Nalge Nunc) for 20−22 h. They were then cultured in maturation medium without dbcAMP or hormones for an additional 24 h. Maturation culture was carried out at 39 °C under an atmosphere of 5% CO_2_, 5% O_2_, and 90% N_2_. Only oocytes displaying the first polar body were deemed mature oocytes.

IVM was performed according to the previous report [43] for the bovine ovarian COCs prepared as described above. Approximately 10–15 COCs were cultured in 100 μL drops of Medium 199 supplemented with Earle’s salts, 25 mM Hepes, 10% (v/v) FBS, 0.02 U/mL FSH, and 0.5 mM sodium pyruvate. The COCs were then incubated under paraffin oil in an atmosphere of 5% CO_2_ for 18−22 h at 38.5 °C.

Cumulus cells were then removed from the porcine and bovine COCs by digestion with 150 IU/mL hyaluronidase for 5 min followed by gentle pipetting. Cumulus-free oocytes displaying the first polar body were collected under a stereomicroscope and used as mature oocytes.

### 2.6. Assessment of Homologous and Heterologous Binding of Porcine and Bovine Sperm to the ZP

Cryopreserved porcine and bovine sperm were thawed at 37 °C and washed twice with modified Krebs-Ringer bicarbonate solution (mKRB) [16]. The sperm were resuspended in mKRB and incubated at 37 °C for 30 min (porcine sperm) or 3 min (bovine sperm) under an atmosphere of 5% CO_2_ in air. The sperm suspension (10 µL) was added to 10−20 immature or mature porcine or bovine oocytes in mKRB (60 µL) containing 2 mM caffeine. The final concentration of sperm was 2 × 10^6^ cells/mL, and the mixture was incubated at 37 ×C for 3 h under paraffin oil. The oocytes were washed 10 times by transferring them into fresh mKRB using a wide-bore pipette (200 µm) and then fixed for 30 min with 3% glutaraldehyde in PBS. The bound porcine and bovine sperm heads were stained with Hoechst 33342 and counted under a fluorescence microscope.

### 2.7. Indirect Immunofluorescent Labeling of Porcine Oocytes

Immature and mature porcine oocytes were incubated for 10 min at 25 °C with rabbit anti-porcine ZP2 (1:50) [16]. After washing three times with fresh 0.5% BSA/PBS, the oocytes were incubated for 10 min at 25 °C in the dark with Alexa 546-conjugated goat anti-rabbit IgG (Molecular Probe, Eugene, OR) in 0.5% BSA/PBS. After washing three times with 0.5% BSA/PBS, the oocytes were observed under a confocal laser-scanning microscope (BA560-600; Olympus, Tokyo, Japan) equipped with an Alexa 546 filter (excitation wavelength, 543 nm). 

### 2.8. Lectin Blotting and Western Blotting for Porcine ZPGs

Heat-solubilized ZPGs from immature and mature porcine oocytes were separated by SDS-PAGE using a 12.5% polyacrylamide gel under nonreducing conditions. The proteins were transferred to polyvinylidene difluoride membranes (Millipore, Bedford, MA) and subjected to lectin blotting with biotin-conjugated GNA lectin as described previously [6]. The membranes were stripped with a buffer containing 62.5 mM Tris–HCl, 2% SDS, and 0.1 M β-mercaptoethanol (pH 6.8) at 50 °C for 30 min and then subjected to Western blot analysis with an anti-porcine ZP2 as described previously [16].

### 2.9. Preparation of Acrosome-Reacted Sperm and Zona-Free Oocytes

Cryopreserved porcine sperm were thawed at 37 °C, washed twice in BSA-free Peterson's medium, and then centrifuged at 600 × *g* for 2 min. The pellets were suspended in the same medium and mixed with the ionophore A23187 to a final concentration of 10 μM. The suspension was incubated at 37 °C for 5 min under an atmosphere of 5% CO_2_ in air and centrifuged at 600 × *g* for 2 min. After washing twice, the sperm pellet was resuspended in medium at a final concentration of 1.2 × 10^7^ cells/mL. Acrosomal status was assessed by cytochemical staining with rhodamine-conjugated *Arachis hypogaea* agglutinin (PNA, Seikagaku Co.) [44]. Stained sperm were observed under a BH2-QRFL fluorescence microscope (Olympus). Sperm showing fluorescence in the acrosomal region were designated acrosome-intact, whereas those without acrosomal fluorescence were designated acrosome-reacted. Before ionophore treatment, approximately 10% of sperm were acrosome-reacted; this value rose to 72% after ionophore treatment.

Cryopreserved bovine sperm were thawed at 37 °C and washed twice with BSA-free Peterson's medium containing 10 mM CaCl_2_. The pellet was resuspended in the same medium, treated with the ionophore A23187 (final concentration, 20 μM), and the suspension was incubated further at 37 °C for 90 min. Acrosomal status was assessed by cytochemical staining with fluorescein-conjugated *Pisum sativum *agglutinin (PSA, Sigma) [45]. Before ionophore treatment, approximately 13% of sperm were acrosome-reacted, which increased to 69% after ionophore treatment.

Zona-free porcine and bovine oocytes were prepared from oocytes subjected to IVM. The oocytes were suspended in a pronase/PBS solution at a concentration of 1 mg/mL for porcine oocyte or 5 mg/mL for bovine oocyte, and then incubated at 37 °C for 1 min. The thin residual layers were physically removed using a narrow bore pipette. Complete removal of the ZP was confirmed by indirect immunofluorescent staining with anti-porcine ZP4 (ZPB) antibody [46].

### 2.10. Binding Assay for A23187-Treated Sperm and Glycolipid Analogs

The binding assay for the A23187-treated porcine and bovine sperm, the pretreatment of sperm with the appropriate ZPG mixture, and the pretreatment with trypsin were performed as described above for acrosome-intact sperm.

### 2.11. Binding Assay for Homologous and Heterologous A23187-Treated Sperm and Zona-Free Oocytes

Five to 10 porcine or bovine zona-free oocytes were placed in 60-µL drops of A23187-treated sperm suspension in mKRB (2 × 10^6^ cells/mL). The drops were incubated at 37 °C for 3 h under an atmosphere of 5% CO_2_ in air. To remove loosely attached sperm, the zona-free oocytes were transferred 10 times into fresh mKRB using a narrow bore pipette. The oocytes were fixed with 3% glutaraldehyde for 30 min, stained with Hoechst 33342, and the number of bound sperm heads was counted under a fluorescence microscope. 

### 2.12. Lectin Labeling

Immature, mature, and zona-free mature oocytes were suspended separately in 30-µL drops of 0.5% BSA/PBS containing biotin-conjugated lectin at a concentration of 1 µg/mL The following lectins of their corresponding carbohydrate-binding specificities were used: RCA, β-D-Gal; GNA, α-D-Man; WGA, β-D-GlcNAc/Neu5Ac; WFA, D-GalNAc; SSA, Neu5Acα2-6Gal/GalNAc; MAM, Neu5Acα2-3Galβ1-4GlcNAc; AAL, α-L-Fuc. The oocytes were incubated at room temperature for 10 min and then washed three times in fresh 0.5% BSA/PBS. The washed oocytes were transferred into 30-µL drops of UltraAvidin–Fluorescein (1 µg/mL in PBS) and incubated in the dark for 5 min at room temperature. After washing an additional five times, the oocytes were placed under a confocal laser-scanning microscope (FV-300; Olympus, Tokyo, Japan) equipped with an FITC filter (Olympus BA510IF; excitation wavelength, 488 nm). The vertical-sectional fluorescence views were monitored in porcine oocytes labeled with GNA and in bovine oocytes labeled with RCA. The equatorial region on each fluorescent cell was selected and the mean fluorescence intensity/area for the region was determined using the Fluoview software (Olympus).

### 2.13. Statistical Analysis

Student's *t*-test or Welch's *t*-test was applied to determine statistical differences between the two groups using a signficance level of *p *< 0.05.

## 3. Results and Discussion

### 3.1. Binding of Porcine and Bovine Sperm to Glycolipid Analogs

Fewer than three porcine or bovine sperm were found in 0.1 µL of the sperm suspension recovered from wells without glycolipid analog or ZPG mixture, whereas 43 porcine and 33 bovine sperm were found in 0.1 µL of the sperm suspensions recovered from wells coated with homologous ZPG mixture. These results indicate that low, nonspecific binding to the plastic plates occurred. The binding affinities of the porcine and bovine sperm for the glycolipid analogs were scored and expressed as the percentage of the number of sperm bound to the homologous ZPG mixture of each assay and then combined (Figure 2), because the number of sperm bound to the homologous ZPG mixture varied (28–61 for porcine; 25–47 for bovine). The number of porcine sperm bound to β-Gal-C6BDB was significantly higher than the number bound to β-GalNAc-C6BDB (*p* < 0.05) (Figure 2A). The number of porcine sperm bound to β-GalNAc-C6BDB was significantly higher than the number bound to α-Man-C6BDB or to β-Glc-C6BDB (*p* < 0.05). The number of porcine sperm bound to α-Man-C6BDB and β-Glc-C6BDB were not significantly different. The number of bovine sperm bound to α-Man-C6BDB was significantly higher than the number bound to β-Glc-C6BDB or β-GlcNAc-C6BDB (*p* < 0.05) (Figure 2B). The binding of porcine sperm to β-Gal and β-GalNAc as well as to the porcine-ZPG mixture was markedly reduced after the pretreatment with the ZPG mixture (open columns in Figure 2A). The binding of bovine sperm to α-Man and β-GlcNAc as well as to the bovine-ZPG mixture also decreased markedly after pretreatment with the bovine-ZPG mixture (open columns in Figure 2B). The acrosomal status of the sperm was observed by chlortetracycline staining [47]. The percentages of capacitated, acrosome-intact sperm and acrosome-reacted sperm were approximately 70% and 2%–4%, respectively, for porcine and bovine sperm. The acrosome reaction was not induced by incubating the porcine and bovine sperm with homologous ZPG mixture in the Peterson's medium [7]. Cryopreserved mammalian sperm are in a state resembling capacitation, and thawed epidiymal porcine sperm possess fertilizing capacity without preincubation [48,49,50]. When plastic wells were coated with OH-C6DBD, which lacked a sugar moiety, and then subjected to the sperm-binding assay, fewer than three porcine or bovine sperm were found in 0.1 µL of the suspension, indicating that the sperm showed affinity for the sugar moiety but not for the alkyl chain.

Thus, bovine sperm bound to α-Man-C6BDB (Figure 2B); however, in an *in vitro* competition assay, α-methylmannoside (22 mM) did not inhibit bovine sperm-ZP binding [17]. In the present study, a solution of glycolipid analog (0.1 mM) was added to each well and the solvent alcohol was evaporated off, resulting in a two-dimensional carbohydrate coating. Because one-tenth the amount of glycolipid analog (0.01 mM) did not exhibit binding activity in the plate assay, our results showed that the density of the carbohydrate residue is a critical factor for binding activity. In pigs and cattle, neither ZP3 nor ZP4 shows binding activity for sperm but heterocomplex of ZP3/ZP4 shows the activity, suggesting that the valency of nonreducing terminal residues and the conformation of protein skeletons are important for maximal sperm ligand activity [5,6,7]. The present results are consistent with this idea.

The carbohydrate-binding specificities of acrosome-intact porcine and bovine sperm revealed in this study are consistent with our previous results, which showed that the nonreducing terminal β-Gal residues and the nonreducing terminal α-Man residues of the *N*-linked carbohydrate chains on ZPGs play essential roles in porcine and bovine sperm binding, respectively (Figure 1A,B) [17,18]. The present results are also consistent with the recent report that porcine sperm bind to the nonreducing terminal β-Gal residues of rabbit erythrocytes but bovine sperm do not [51].

Trypsinization significantly reduced sperm binding to the glycolipid analogs and to the ZPG mixture (*p* < 0.05) (Figure 2C). The percentages of capacitated, acrosome-intact sperm and acrosome-reacted sperm were not affected by trypsin treatment. When trypsin was inhibited with tosyl-L-lysylchloromethylketone, the binding of sperm to the glycolipid analogs and to the ZPG mixture did not decrease (data not shown). These results indicate that a large part of the carbohydrate-binding moieties on the surface of the sperm consists of proteins. Then, the proteins are referred to as carbohydrate-binding proteins (CBPs) in the remainder of this article. Porcine CBPs showed strong affinities for β-Gal and β-GalNAc, whereas bovine sperm had stronger affinities for α-Man, β-Glc, and β-GlcNAc (Figure 2), suggesting that CBP-binding affinities are dependent on the configuration of the 3-OH and 4-OH groups on nonreducing terminal sugars. That is, the β-Gal and β-GalNAc residues contain an equatorial 3-OH and an axial 4-OH, whereas the α-Man, β-Glc, and β-GlcNAc residues contain an equatorial 3-OH and an equatorial 4-OH. The 3-OH and 4-OH configurations have been reported to be indispensable for the binding of mannose to C-type animal lectins [52,53]. Treating porcine and bovine sperm with species-specific ZPG mixtures virtually eliminated the affinity of these CBPs for their preferred sugar residues (Figure 2), indicating that the CBPs on acrosome-intact sperm are involved in the binding of sperm to ZPGs in both species.

### 3.2. Homologous and Heterologous Binding of Porcine and Bovine Sperm to ZP

**Figure 3:**
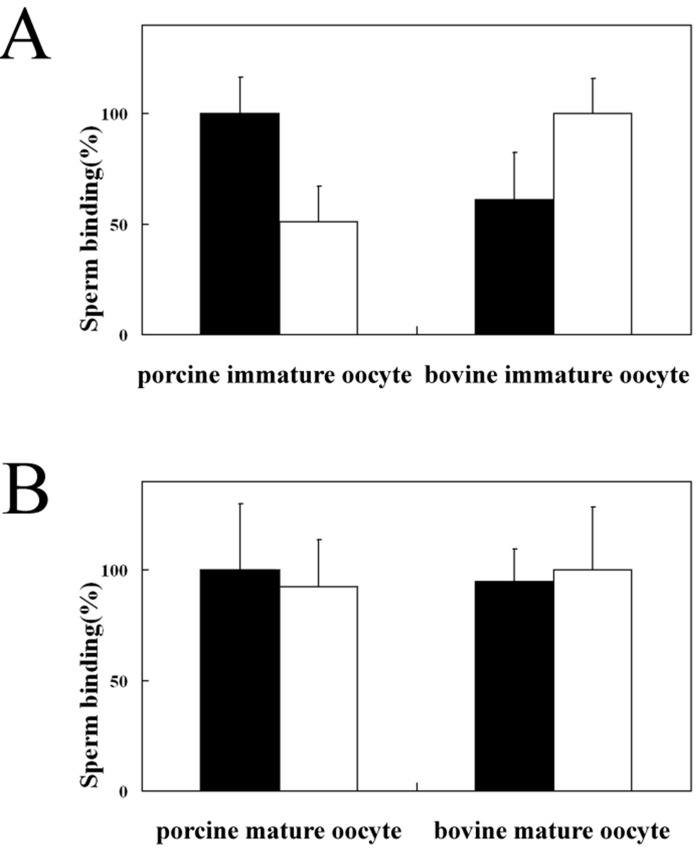
Homologous and heterologous sperm–oocyte binding in the pig and bovine. (**A**) The number of homologous acrosome-intact sperm bound toimmature oocytes is expressed as 100%. (**B**) The number of homologous acrosome-intact sperm bound tomature oocytes is expressed as 100%. The mean number of heterologous sperm bound to oocytes is expressed as a percentage of the number of bound homologous sperm. Assays were repeated three times, and the data are shown as the mean ± SD. Solid bars, porcine sperm; open bars, bovine sperm.

The mean number of porcine sperm bound to immature porcine zona-intact oocytes was 200.9, whereas the mean number of bovine sperm bound to immature porcine zona-intact oocytes was 104.3. In contrast, the mean number of bovine sperm bound to immature-bovine, zona-intact oocytes was 380.5, whereas the mean number of porcine sperm bound to the same oocytes was 230.2. Thus, in both porcine and bovine oocytes, the number of heterologous sperm bound to oocytes was about half of that of homologous sperm bound to oocytes (Figure 3A). This indicates that the interaction between sperm and the ZP of immature oocytes is species-selective (*p *< 0.001). This observation is consistent with the previous report that porcine sperm binds to bovine zona-encased oocyte [34]. We obtained similar results using dead immature oocytes collected from cryopreserved ovaries (data not shown). The reason why the number of porcine sperm bound to immature bovine oocytes was larger than that of porcine sperm bound to immature porcine oocytes is unclear. The binding selectivity decreased markedly after oocyte maturation; the number of bovine sperm bound to mature, porcine oocytes (132.9) was similar to the number of porcine sperm bound to mature, porcine oocytes (144.7) (Figure 3B, *P* > 0.1). Furthermore, the number of porcine sperm bound to mature bovine oocytes (291.6) was also similar to the number of bovine sperm bound to mature bovine oocytes (315.7) (Figure 3B, *P* > 0.1). In both porcine and bovine oocytes, the number of homologous sperm bound to oocytes decreased after maturation and the number of heterologous sperm bound to oocytes increased after maturation.

### 3.3. Lectin Staining in Immature and Mature Oocytes

**Figure 4:**
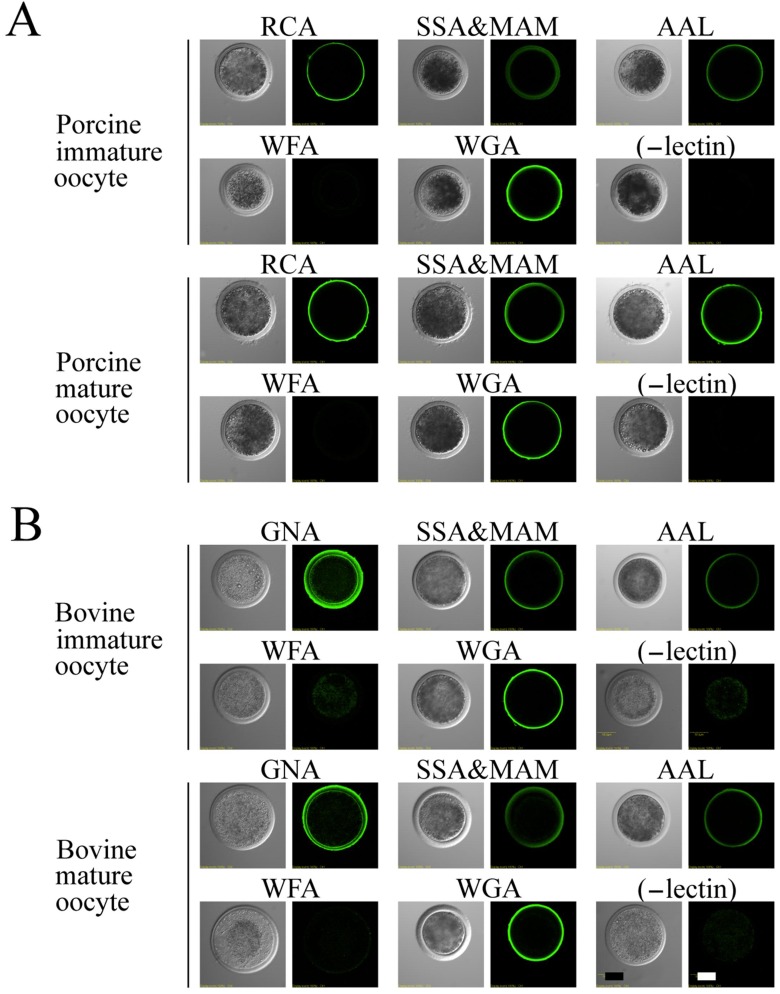
Localization of biotin-conjugated lectins in the porcine and bovine zona pellucida. The porcine (**A**) and bovine (**B**) immature and mature oocytes were incubated separately in BSA/PBS containing biotin-conjugated lectin(s) or without lectins (-lectin) followed by washing and incubation with UltraAvidin–Fluorescein. Left panels, phase-contrast; right panels, fluorescence. Scale bar = 50 µm.

**Figure 5:**
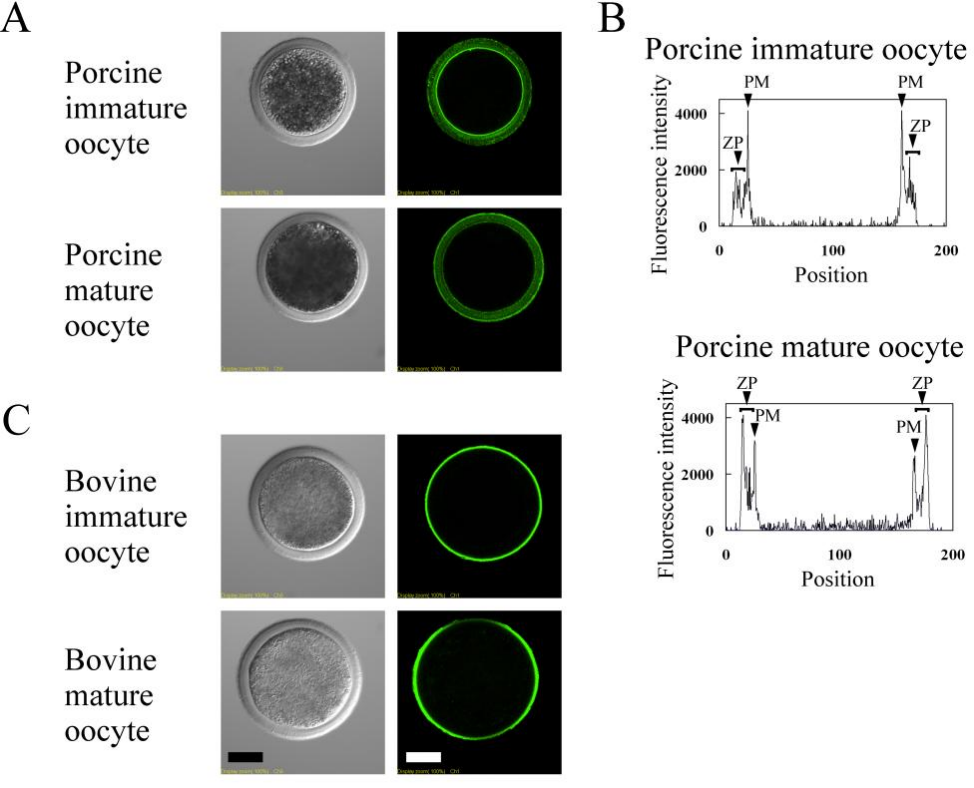
Localization of biotin-conjugated GNA-lectin in the porcine zona pellucida (**A** and **B**) and of RCA-lectin in the bovine zona pellucida (**C**). Left panels in A and C, phase-contrast; right panels in A and C, fluorescence. In the pig, the vertical sectional fluorescence intensities (arbitrary scale) are also shown (**B**). ZP, zona pellucida; PM, plasma membrane. Scale bar = 50 µm.

We investigated the localization of the nonreducing ends of the carbohydrate chains on ZPGs of immature and mature oocytes by using biotin-conjugated lectins followed by FITC-avidin staining (Figure 4 and Figure 5), because the nonreducing ends of neutral *N*-linked chains on ZPGs are involved in sperm binding to the ZP in pigs and cattle [16,17,18], and the sperm-binding selectivity of porcine and bovine oocytes changed after maturation.

In the porcine ZP, positive staining occurred in immature and mature oocytes after labeling with RCA, WGA, and AAL (Figure 4A). Weak staining of SSA/MAM occurred in immature oocytes, which intensified after maturation, indicating increase in sialylation during maturation. Previous studies demonstrated that maturation involves an increase in the acidity of ZPGs. No staining took place with WFA labeling. *O*-linked chains of porcine ZP contain nonreducing terminal GalNAc residues [54,55], but in the present study, we did not detect GalNAc residues, probably due to the presence of a small amount of GalNAc. GNA staining was weak throughout the ZP of immature oocytes but concentrated in the outer regions of the ZP in mature oocytes (Figure 5A,B). With the exception of α-Man, these results agree with our previous structural analyses of the *N*-linked carbohydrate chains of porcine ZP3 and ZP4 [56,57] and show that β-Gal, α-Man, β-GlcNAc, and α-sialic acid were present at the nonreducing ends of the porcine ZPGs in both immature and mature oocytes (Figure 4).

In bovine oocytes, the most intense staining was observed with GNA on the outer surfaces of both immature and mature oocytes (Figure 4B). Furthermore, staining was observed with WGA, AAL, and SSA/MAM labeling. Similar to porcine oocytes, no staining was observed after WFA labeling. Although no changes occurred in the distribution of carbohydrate chains, the fluorescence intensity of the RCA-lectin labeling increased in mature oocytes compared to immature oocytes (Figure 5C). These results support our results of a previous structural analysis of *N*-linked carbohydrates present in mixed-bovine ZPGs [58].

### 3.4. Localization of ZP2 in Porcine Oocytes

**Figure 6:**
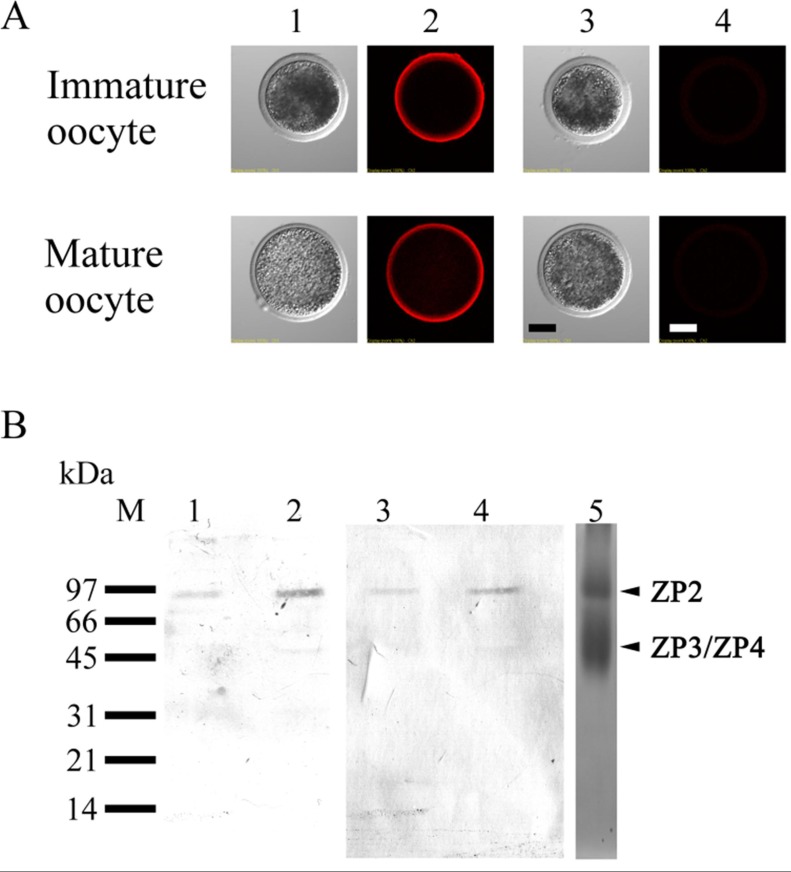
Localization of porcine ZP2 in immature and mature oocytes. (**A**) Indirect immunostaining of porcine immature and mature oocytes using anti-porcine ZP2 (panels 1 and 2), and without antibody (panels 3 and 4). Panels 1 and 3, phase-contrast; panels 2 and 4, fluorescence. (**B**) GNA-lectin blots (lanes 1 and 2) and Western blots using anti-porcine ZP2 (lanes 3 and 4) of porcine-zona protein mixtures from immature (lanes 1 and 3) and mature (lanes 2 and 4) oocytes. Silver staining of immature, porcine-zona protein mixture prepared from cryopreserved ovaries (lane 5) shows the positions of ZP2, ZP3, and ZP4. Scale bar = 50 µm.

High-mannose type chains are only found on ZP2 (also referred to as ZPA) in pigs [59,60,61]. Therefore, we examined if the distribution of ZP2 changed after maturation. Indirect immunostaining with anti-ZP2 indicated that ZP2 is distributed throughout the ZP of immature porcine oocytes (Figure 6A). Neither fluorescence intensity nor distribution of ZP2 changed after maturation.

We detected nonreducing terminal α-Man residues on porcine ZPGs from immature and mature oocytes using biotin-conjugated GNA lectin to further investigate why the distribution of nonreducing terminal α-Man residues changed after maturation. Porcine ZPGs obtained from cryopreserved ovaries were subjected to SDS-PAGE under nonreducing conditions. Silver staining (lane 5) revealed bands of 90 kDa (ZP2) and 55 kDa (ZP3 and ZP4) (Figure 6B, lane 5), as shown in previous studies [62]. Due to a limited number of available oocytes, the ZPGs from immature and mature porcine oocytes were not silver-stained but were subjected to GNA-lectin (Figure 6B, lanes 1 and 2, respectively) and Western blotting using anti-porcine ZP2 (Figure 6B, lanes 3 and 4, respectively). The results from both blots showed a single band in the ZPs from immature and mature oocytes. Thus, the α-Man residues were detectable on ZP2 but not on ZP3 or ZP4, which was consistent with the previous reports [59,60,61]. These results indicate that the change in the distribution of nonreducing terminal α-Man residues after maturation was not due to redistribution of ZP2 to the outer surface of the ZP or to modification of ZP3 and ZP4 carbohydrates.

Previous studies demonstrated that maturation involves both biochemical and functional alterations to the ZP in porcine oocytes [60,61,63,64], including an increase in the acidity of ZPGs. These changes may induce the conformational change of ZPGs and facilitate the exposure of ZP2 carbohydrate chains, resulting in an increased number of bovine sperm bound to mature porcine oocytes. We observed increased RCA-lectin labeling during maturation of bovine oocytes, although we found no change in the localization of bovine ZP sugar residues to which lectins were accessible (Figure 5C). As a result, an increase occurred in the number of porcine sperm bound to the bovine ZP. These results demonstrate that the specificity and variety of CBPs on the surface of acrosome-intact sperm, the changes in the sperm binding to oocytes after maturation, and the changes in the lectin-staining patterns of the ZP after maturation are consistent with each other. Thus, these results support the hypothesis that the interaction between the CBPs on the sperm and the carbohydrates on the ZP mediates the binding of sperm to the ZP. Our results further revealed that the specific interaction between CBPs and ZPGs is not responsible for species selectivity during heterologous *in vitro* incubation between porcine and bovine gametes.

### 3.5. Binding of A23187-Treated Porcine and Bovine Sperm to Glycolipid Analogs

**Figure 7:**
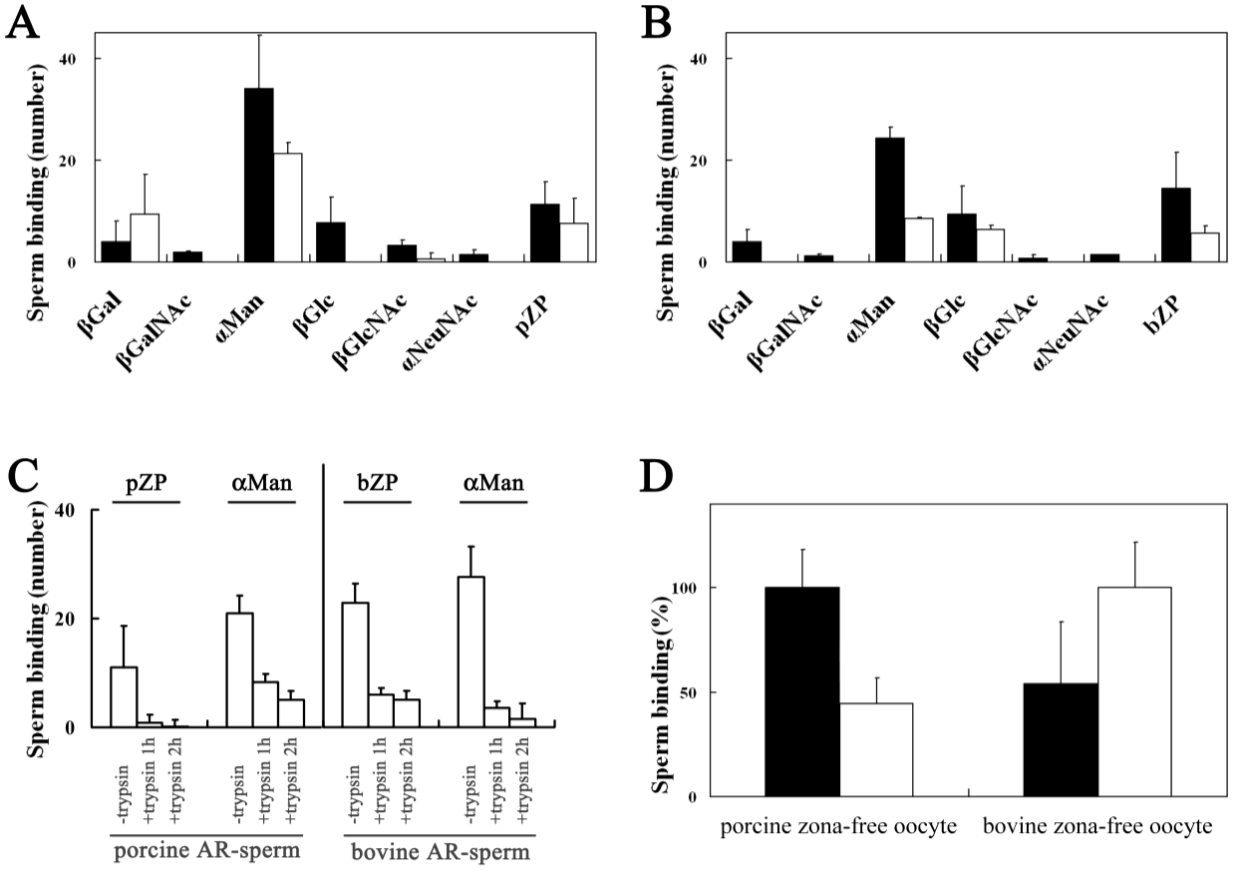
Binding of A23187-treated sperm to the glycolipid analogs and to zona-free oocytes. The number of A23187-treated porcine sperm (**A**) and bovine sperm (**B**) that bound to plastic wells coated with glycolipid analogs or porcine-zona protein mixture (pZP) and the bovine-zona protein mixture (bZP). The sperm samples included the A23187-treated sperm without preincubation with the zona protein mixture (solid bars). For the glycolipid analogs of βGal, αMan, and βGlcNAc, and for pZP in (**A**) and for those of αMan, and βGlc, and for bZP in (**B**), the numbers of A23187-treated sperm that were treated with the zona protein mixture, and bound, are also shown (open bars). The assays were repeated three times, and the data are shown as the mean ± SD. βGal, β-Gal-C6BDB; βGalNAc, β-GalNAc-C6BDB; αMan, α-Man-C6BDB; βGlc, β-Glc-C6BDB; βGlcNAc, β-GlcNAc-C6BDB; αNeuNAc, α-NeuNAc-C6BDB. (**C**) Effect of trypsinization for 1 or 2 h of sperm on the binding of the A23187-treated (AR-sperm) porcine and bovine sperm to the zona protein mixture (pZP or bZP, respectively) or to the glycolipid analog α-Man. (**D**) Homologous and heterologous binding of the A23187-treated sperm to porcine and bovine zona-free oocytes. The mean number of the heterologous A23187-treated sperm bound to the zona-free oocytes is expressed as a percentage of the mean number of homologous sperm bound to the zona-free oocytes (shown as 100%). Assays were repeated three times, and the data are shown as the mean ± SD. Solid bars, porcine sperm; open bars, bovine sperm.

The acrosome reaction was induced in approximately 70% of the motile, porcine and bovine sperm treated with A23187. The number of A23187-treated sperm that bound to the glycolipid analogs is shown in Figure 7. The number of A23187-treated porcine sperm bound to α-Man-C6BDB was significantly higher than the number bound to β-Gal-C6BDB or to the porcine-ZPG mixture (*p* < 0.05) (Figure 7A). Incubation of A23187-treated porcine sperm with the ZPG mixture significantly reduced (*p* < 0.05) binding to α-Man-C6BDB or to the porcine-ZPG mixture (Figure 7A, open bars), but the inhibition was weak compared to the inhibition of the ZPG mixture for the binding of acrosome-intact porcine sperm to β-Gal-C6BDB or to the porcine-ZPG mixture (Figure 2A). These results demonstrated that the carbohydrate-binding components of acrosome-intact porcine sperm and A23187-treated porcine sperm differ in their carbohydrate-binding specificity and their ability to bind to the ZPG mixture.

The A23187-treated bovine sperm exhibited significant binding to α-Man-C6BDB, β-Glc-C6BDB, and the bovine-ZPG mixture (Figure 7B). The number of A23187-treated bovine sperm bound to α-Man-C6BDB was significantly higher than the number bound to β-Glc-C6BDB or to the bovine ZPG mixture (*p* < 0.05). Thus, A23187-treated and acrosome-intact bovine sperm showed similar specificities for certain carbohydrate groups (Figure 7B and Figure 2B, respectively). While A23187-treated and acrosome-intact bovine sperm showed a similar degree of binding to α-Man-C6BDB, the number of sperm bound to the bovine-ZPG mixture was reduced to about 50% after treatment with A23187. Inhibition of the binding of A23187-treated bovine sperm to α-Man-C6BDB and to the bovine-ZPG mixture by preincubation with the bovine-ZPG mixture was significant, and slightly weaker, compared to the inhibition of the binding of acrosome-intact bovine sperm to α-Man-C6BDB and to the bovine-ZPG mixture (Figure 7B, open bars). These results indicate that the carbohydrate-binding components of acrosome-intact bovine sperm and A23187-treated bovine sperm are similar in carbohydrate-binding specificity but differ in their ability to bind to the ZPG mixture. 

The affinities of both porcine and bovine A23187-treated sperm for α-Man-C6BDB and their species-specific, ZPG mixtures were markedly reduced by trypsinization (Figure 7C). When trypsin was inhibited with tosyl-L-lysylchloromethylketone and then used in the assays, the binding of sperm to the glycolipid analogs and to the ZPG mixture did not decrease (data not shown). These results indicate that a large part of carbohydrate-binding moieties on the surface of A23187-treated sperm consisted of proteins.

The carbohydrate-binding specificity of porcine sperm changed from β-Gal to α-Man after treatment with the ionophore A23187, whereas the ionophore treatment did not affect the specificity of bovine sperm (Figure 7A and 7B). In both species, the binding of sperm to α-Man decreased markedly after trypsinization of sperm, indicating that these carbohydrate-binding moieties consist mainly of proteins (Figure 7C). These results suggest that a C-type mannose-binding lectin-like CBP is predominant on acrosome-reacted porcine sperm as well as on acrosome-reacted bovine sperm. In both species, the CBPs on acrosome-reacted sperm had a stronger affinity for α-Man than for the ZPG mixture. Acrosome-reacted sperm treated with the ZPG mixture had a smaller decrease in binding affinity for the glycolipid analogs and the ZPG mixture than for the acrosome-intact sperm (Figure 7 and Figure 2, respectively). This suggests that the CBPs on acrosome-reacted porcine and bovine sperm do not play major roles in the binding of sperm to the ZPG mixture compared to acrosome-intact sperm. Whether secondary binding of acrosome-reacted porcine and bovine sperm to the ZPGs requires the intact supramolecular structure of the zona matrix remains to be clarified.

### 3.6. Homologous and Heterologous Binding of A23187-Treated Sperm to Zona-Free Oocytes

We assessed the binding affinity of A23187-treated sperm to zona-free oocytes. In both species, the zonae were removed completely by pronase digestion, which was confirmed by indirect immunofluorescent staining with anti-porcine ZP4 cross-reactive with bovine ZP4 [46] (data not shown). The mean number of A23187-treated porcine and bovine sperm bound to the homologous porcine and bovine oolemma were 100.4 and 126.1, respectively. The number of A23187-treated bovine sperm that bound to pig oolemma, and vice versa, was approximately 50% of the number that bound homologously (Figure 7D). Thus, although A23187-treated sperm−oocyte binding is not species-specific, it is species-selective (*p *< 0.001). 

### 3.7. Lectin Labeling in Zona-Free Mature Oocytes

**Figure 8:**
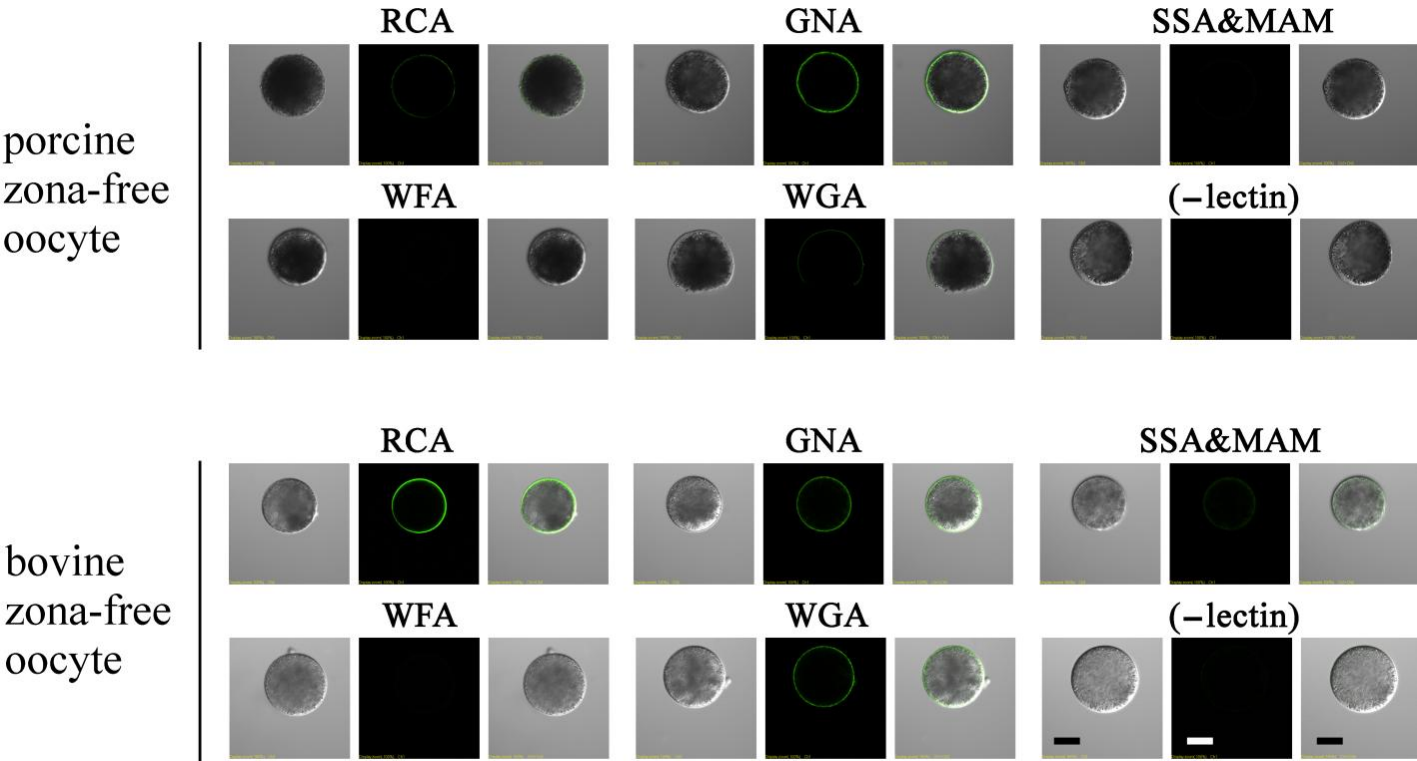
Localization of biotin-conjugated lectins in porcine and bovine zona-free oocytes. The oocytes were incubated separately with lectin (indicated above each panel) or without lectins (-lectin) followed by washing and incubation with UltraAvidin–Fluorescein. Left panels, phase-contrast; middle panels, fluorescence; right panels, phase-contrast and fluorescence images merged. Scale bar = 50 µm.

GNA labeling of mature, porcine zona-free oocytes revealed more intense staining than with the other lectins tested (Figure 8). We observed significant, but weak, staining after RCA or WGA labeling, but no staining occurred using SSA/MAM or WFA labeling. In contrast, intense staining was observed after RCA, GNA, and WGA labeling in mature bovine zona-free oocytes and significant, but weak, staining was seen after SSA/MAM labeling (Figure 8). No staining occurred in mature bovine zona-free oocytes after WFA labeling.

In pigs and cattle, we observed significant species selectivity between acrosome-reacted sperm and the oolemma (Figure 7D). As membrane glycolipids generally lack the α-Man residue, the results presented in Figure 8 suggest that high-mannose-type carbohydrate chains are present on porcine and bovine-oolemmal glycoproteins. CBPs on the membrane of porcine and bovine acrosome-reacted sperm likely bind to high-mannose-type chains on oolemmal glycoproteins (Figure 8). We found similar carbohydrate-binding specificities for acrosome-reacted porcine sperm and for acrosome-reacted bovine sperm. Furthermore, the positive GNA-lectin staining of the porcine and bovine oolemma did not explain the species-selective binding between acrosome-reacted sperm and the oolemma. Taken together, these results suggest that the interaction between CBPs on the acrosome-reacted sperm and the glycoprotein–carbohydrate moieties on the oolemma may mediate part of the interaction between gametes; however, other components may be involved in species-selective interactions between sperm and oolemma in pigs and cattle.

## 4. Conclusions

Sperm and egg morphology may affect sperm–oocyte recognition [65]. Porcine and bovine gametes are suitable materials for the analysis of heterologous interactions, as their sperm have a similar dish-shaped head about 9 µm in length and 4.5 µm in width, and their oocytes are almost the same size (about 150 µm in diameter). In IVF, complete species specificity was observed between heterologous gametes in pigs and cattle (Naoto Yonezawa and Minoru Nakano, unpublished data). Although the results supported that sperm−oocyte binding is a carbohydrate-mediated event, they did not establish species specificity. Acrosome-intact sperm surface components, such as lectins, glycosyltransferases, glycosidases, and other carbohydrate-binding components, have been described in various mammals; however, very little information is available regarding the proteinaceous components that bind oolemmal carbohydrates on the surface of acrosome-reacted sperm [25]. To better understand the initial phase of fertilization, further studies are required to characterize the CBPs present on acrosome-reacted as well as acrosome-intact sperm.
